# Placement Poverty in Rural Health Training: A Persistent Structural Barrier

**DOI:** 10.1111/ajr.70246

**Published:** 2026-08-31

**Authors:** Astrid C. Turner, Kathryn W. Fitzgerald, Rachel Morris, Sandra Thompson

**Affiliations:** ^1^ Western Australian Centre for Rural Health (WACRH) Geraldton Western Australia Australia

## Abstract

**Aim:**

This commentary explores the growing financial challenges associated with rural and remote clinical placements for nursing and allied health students and considers their implications for workforce development in regional Australia. Drawing on routinely collected survey data provided by the Western Australian Centre for Rural Health (WACRH) between 2017 and 2024, we use student‐reported experiences as evidence of persistent concerns about financial burdens during rural clinical placements and discuss potential policy and practice responses.

**Context:**

Rural workforce shortages remain a persistent national challenge for University Departments of Rural Health which facilitate rural training experiences for health science students. Rising costs of living, housing shortages across Australia, and a policy shift towards longer rural placements have increased concerns about placement poverty, a term used to describe the financial burden students experience because of completing mandatory professional placements. WACRH includes support for students undertaking clinical placements in our regions through accommodation at no personal cost to the student, vehicle access for placement activities and limited travel subsidies to remote sites.

**Approach:**

Data from pre‐ and post‐placement surveys (2017–2024) was analysed. Quantitative data from Likert‐style questions on financial concerns were analysed in Stata, while qualitative open‐ended responses describing financial challenges were manually coded. Analyses examined variation by year and location.

**Conclusion:**

Financial concerns have increased over time, with a notable rise in students reporting being “Concerned” or “Very concerned” since 2022. Many students reported significant cost‐of‐living pressures, dual accommodation expenses, additional food and travel costs, and limited financial support. Achieving national rural workforce policy outcomes requires greater support for rural placements, including funding that reflects the true costs of rural training.

The long‐standing wicked problem of recruitment and retention of health professionals in rural and remote (hereafter just termed rural) areas has required innovative and long‐term approaches to seek solutions. These include encouraging rural residents into rural health careers and encouraging health graduates to consider working rurally [1]. There are evident advantages to shaping the hearts and minds of health science students in training to consider working rurally [2, 3]. The Australian government has over many years funded University Departments of Rural Health (UDRHs) to provide academic and logistical support for students to undertake training in rural areas. The models of support vary across UDRHs, with the Western Australian Centre for Rural Health (WACRH)—whose head office is in Geraldton in the Midwest region of Western Australia (WA)—providing support and clinical placements for nursing and allied health students [4]. Most WACRH students come from metropolitan areas and are enrolled in Perth‐based universities.

A persistent challenge for UDRHs has been reticence by students to undertake rural placements, with financial burden one reason noted [5, 6, 7]. The term “placement poverty” has more recently come into common usage and been defined as the financial burdens imposed upon students by the completion of mandatory professional placement [8]. This reflects rises in the cost of living, with rural placement students reporting additional costs associated with leaving their family or existing accommodation, giving up, at least temporarily, the employment that supports their living expenses while studying [6, 8, 9]. Student financial concerns have been given much greater prominence in recent times when the cost of living in Australia is often framed as a crisis, with headlines and public attention given to the issue [10, 11].

There is also a strategic approach by funders and UDRHs to support longer rural placements, informed by evidence showing a consistent association between rural placement exposure and later rural practice intention [2] and clear thresholds indicating that students with more than three rural placements or over 16 cumulative placement weeks are significantly more likely to consider rural employment [12].

To support students and reduce their costs to go rural, WACRH provides accommodation at no personal cost to the student in each of its locations. Financial assistance from WACRH for travel to and from the remote regions of the Pilbara and Gascoyne is available to most students who undertake a placement of at least four weeks' duration.

As part of supporting students and monitoring our educational program, WACRH asks students to complete a pre‐ and post‐placement survey. A harmonised dataset from 2017 and 2024 was available, based on a student survey, and captured their concerns and placement experiences. We used this longitudinal dataset to explore the question of any changes over time in student concerns about finances.

The survey instruments over this period were distributed via the Qualtrics platform. Quantitative data were analysed using Stata, while qualitative responses were extracted into Excel and analysed manually. Our analysis focused on students' financial concerns and funding sources, with particular attention to variations by year of placement, geographical location, and academic discipline. Open ended responses in the commencement survey related to financial concerns were extracted and provide insights into the concerns of students. Issues related to finances reported in students' reports on “least attractive features” at the end of their placement were also included.

The University of Western Australia's Human Research Ethics Committee approved use of this anonymised data for monitoring, quality improvement, reporting and publication purposes (HREC 2023/ET000289). Students were informed, when they were provided with the survey link, that their de‐identified responses may be used for reporting and dissemination of findings.

At commencement, students were asked: “Please rate your level of concern relating to the following factors: Finances” (response options: Very concerned/Concerned/Neutral/Not very concerned/Not at all concerned). Results are shown in Table 1, with an additional column of the combined data for both Concerned and Very concerned categories, highlighting an increase from 29.6% in 2017 to 38.2% in 2024, with the largest increase occurring after 2021. Across all years, “Neutral” was the largest category (794 responses, 31.8% overall). In the earlier years (2017–2021), “Very concerned” was low (2.6%–5%), and “Concerned” hovered around 18%–24%. Between 2022 and 2024, there was an increase in “Very concerned” (up to 9.4% in 2024) and “Concerned” (peaking at 31.2% in 2023), suggesting increased concern related to finances over time.

**TABLE 1:** Level of financial concerns (and percentage) per year (commencement survey).

		Not at all concerned	Not very concerned	Neutral	Concerned	Very concerned	Combined concerned^a^	Total
2017	*n*	33	75	89	69	14	83	280
	(%)	(11.8)	(26.8)	(31.8)	(24.6)	(5.0)	(29.6)	
2018	*n*	72	113	117	71	16	87	389
	(%)	(18.5)	(29.1)	(30.1)	(18.3)	(4.1)	(22.4)	
2019	*n*	43	80	106	66	8	74	303
	(%)	(14.2)	(26.4)	(35.0)	(21.8)	(2.6)	(24.4)	
2020	*n*	71	89	114	62	9	71	345
	(%)	(20.6)	(25.8)	(33.0)	(18.0)	(2.6)	(20.6)	
2021	*n*	63	109	98	72	10	82	352
	(%)	(17.9)	(31.0)	(27.0)	(20.5)	(2.8)	(23.3)	
2022	*n*	34	65	83	77	9	86	268
	(%)	(13.0)	(24.3)	(31.0)	(28.7)	(3.4)	(32.1)	
2023	*n*	16	43	64	62	14	76	199
	(%)	(8.0)	(21.6)	(32.3)	(31.2)	(7.0)	(38.2)	
2024	*n*	33	67	123	104	34	138	361
	(%)	(9.1)	(18.6)	(34.1)	(28.8)	(9.4)	(38.2)	
Total		365	641	794	583	114	697	2497

*Note:* Pearson *χ* ^2^ = 102.41 Prob = 0.0000, indicates a statistically significant difference in concern levels across years.

^a^ Combined concerned and very concerned.

Around 1 in 4 students report being “Concerned” about finances, with a further 2%–9% reported being “Very concerned” depending on the year. Concern levels vary by location, with larger sites (Geraldton, Karratha, Carnarvon, Port Hedland) showing consistent concern rates around 22%–27%, with “Very concerned” near 4%–6% (detailed site‐level findings are not reported here). While some variation was observed between disciplines, the relatively small subgroup sizes and influence of contextual factors, including placement characteristics, university support and individual circumstances, limit meaningful interpretation of discipline‐specific differences. Typical concerns provided related to financial concerns are in Table 2.

**TABLE 2:** Illustrative student comments related to financial concerns.

*I **will not be able to work** which will stretch my finances and mean I will need to work more during my final semester which may affect my results*.
*I will be **relying on my savings for the duration of placement**. If possible, I will attempt to find a casual job on Sat/Sun mornings (provided it will not interfere with my workload)*.
*I have to continue paying rent and bills whilst in <place> and not earning income*,
***I have to take 2 months off work for placement**, after I finish this one it will be the Christmas and new year's period where my jobs are closed and then I go straight into another 3‐week prac in January*.
*I have no financial support so that is my main stressor—having to pay for things while on prac*.

Student comments noted a lack of upfront clarity over what would be provided on placement, whether and when they might receive scholarship support, where that support may come from, and the time for Centrelink to make changes to enable payments to commence or be increased.

Questions about level of concern about finances while undertaking a placement were not in the end of placement survey; however, some students raised these in response to “What aspects of your rural experience, if any, did you find least attractive?” Comments indicated that some students struggled financially, noting the costs of food and groceries while not earning, the high cost of eating out, dual housing expenses with ongoing costs of rent and bills related to their Perth accommodation, the lack of scholarship support or financial assistance from their university, and petrol and travel costs to get home.

Financial challenges have been reported as a major theme of students' placement journeys, particularly those undertaking rural placements where added costs coincide with social isolation and reduced support systems [5, 12, 13]. We also acknowledge multiple factors can contribute to cost‐of‐living pressures, including post‐pandemic labour market conditions and students' access to casual employment which may have changed over time. However, the elevated cost of everyday living in many rural communities, particularly the higher prices of groceries and essential goods, adds substantially to the financial costs associated with undertaking placements in these locations [14, 15]. These pressures are not distributed evenly; students from lower socioeconomic backgrounds and other marginalised groups often face disproportionate hardship due to limited financial reserves, making them more likely to shorten or opt out of rural or remote placement opportunities [13]. Moreover, the financial strain associated with such placements can generate significant psychological stress and anxiety, with implications for student wellbeing, learning capacity and overall engagement. Thus, promoting longer rural placements to students without adequate income or specific support while on placement is a flawed approach and unlikely to improve willingness to undertake rural placements [12].

Capper et al. argue that while student challenges such as financial stress may arise at the micro (individual) level, their impacts are increasingly visible at the meso (institutional) and macro (national and professional) levels [13]. When these challenges are grouped as cross‐disciplinary barriers, they highlight broader structural issues within healthcare education and placement models.

The Australian Universities Accord prioritises attention to increase opportunities in higher education for equity groups who have hitherto been underrepresented and encourage their admission into university; groups generally of lower socioeconomic status [16]. The Australian Government has implemented a means‐tested practicum payment for health students in midwifery, social work and nursing, and has since announced that the scheme will expand to include additional allied health disciplines from July 2027 [17, 18]. However, the payment is not specific to rural placements, eligibility remains restricted, and students undertaking rural and remote placements often face costs that substantially exceed those experienced in metropolitan settings. As the student responses show, it is being away from their usual home and income from part time work that makes rural placements particularly challenging for these health students. Without adequate recognition of these costs, the severe rural health worker shortages existing in other priority health disciplines (e.g., optometry, dietetics) will also go unaddressed. While the planned expansion is a welcome recognition of placement poverty, additional rural‐specific supports such as accommodation, travel assistance and other targeted measures will remain necessary to ensure equitable access to rural placement opportunities.

Universities are responding, an example being the University of Western Australia (UWA) that has developed a suite of initiatives to support students experiencing costs of living pressures [19] following student feedback and studies highlighting financial hardship among a large proportion of university students that affect their well‐being and academic performance [20, 21]. However, while valuable, these initiatives overwhelmingly benefit on‐campus students and fail to address the additional, often hidden, costs borne by those undertaking rural placements.

There is a strong rationale for extending placement duration and/or distributed cumulative rural placements to enhance students' opportunities to develop core skills in preparation for rural practice. Such experiences and learning allow them to engage meaningfully with rural communities and build connections with health providers at micro, meso and macro levels. However, many health graduate entry students require specific support to cover living costs while on placement, support which currently is not adequately addressed [12, 16, 22, 23]. Our own data indicate that while not all students experience acute privation during placements, many face heightened financial strain, and that concerns about money among those students undertaking rural placements have increased over time. Although the data cannot fully capture the complexity of individual financial circumstances such as parental support or additional university assistance, it contributes to a growing evidence base demonstrating that greater support for students is needed. The need for greater financial support is particularly pertinent as efforts continue to encourage longer or cumulative rural placements to better prepare students for rural practice.

To achieve the desired policy outcomes, increased support for practical placement payments, particularly for those in rural areas, is needed. This must recognise the real costs of rural training and support nursing and allied health students undertaking extended or cumulative rural placements. Without this investment, aspirations to achieve a sustainable rural workforce will remain handicapped rather than being expedited.

## Author Contributions

**Sandra Thompson:** conceptualization (lead), writing – original draft, writing – review and editing (equal). **Astrid C. Turner:** formal analysis (lead), writing – review and editing (equal). **Kathryn W. Fitzgerald:** methodology, writing – review and editing (equal). **Rachel Morris:** methodology, writing – review and editing (equal).

## Conflicts of Interest

The authors declare no conflicts of interest.

## Data Availability

Research data are not shared.
